# Assessment of bacterial endosymbiont diversity in *Otiorhynchus* spp. (Coleoptera: Curculionidae) larvae using a multitag 454 pyrosequencing approach

**DOI:** 10.1186/1471-2180-12-S1-S6

**Published:** 2012-01-18

**Authors:** Jacqueline Hirsch, Stephan Strohmeier, Martin Pfannkuchen, Annette Reineke

**Affiliations:** 1 Geisenheim Research Center, Department of Phytomedicine, Von-Lade-Str. 1, 65366 Geisenheim, Germany; 2 SMS-Development, Ortsstr. 6, 69226 Nussloch, Germany; 3 Center for Marine Research, Institute Ruder Boskovic, Giordano Paliaga 5, 52210 Rovinj, Croatia

## Abstract

**Background:**

Weevils of the genus *Otiorhynchus* are regarded as devastating pests in a wide variety of horticultural crops worldwide. So far, little is known on the presence of endosymbionts in *Otiorhynchus* spp.. Investigation of endosymbiosis in this genus may help to understand the evolution of different reproductive strategies in these weevils (parthenogenesis or sexual reproduction), host-symbiont interactions, and may provide a future basis for novel pest management strategy development. Here, we used a multitag 454 pyrosequencing approach to assess the bacterial endosymbiont diversity in larvae of four economically important *Otiorhynchus* species.

**Results:**

High-throughput tag-encoded FLX amplicon pyrosequencing of a bacterial *16S* rDNA fragment was used to characterise bacterial communities associated with different *Otiorhynchus* spp. larvae. By sequencing a total of ~48,000 PCR amplicons, we identified 49 different operational taxonomic units (OTUs) as bacterial endosymbionts in the four studied *Otiorhynchus* species. More than 90% of all sequence reads belonged either to the genus *Rickettsia* or showed homology to the phylogenetic group of “*Candidatus* Blochmannia” and to endosymbionts of the lice *Pedicinus obtusus* and *P. badii*. By using specific primers for the genera *Rickettsia* and “*Candidatus* Blochmannia”, we identified a new phylogenetic clade of *Rickettsia* as well as “*Candidatus* Nardonella” endosymbionts in *Otiorhynchus* spp. which are closely related to “*Candidatus* Blochmannia” bacteria.

**Conclusions:**

Here, we used multitag 454 pyrosequencing for assessment of insect endosymbiotic communities in weevils. As 454 pyrosequencing generates only quite short sequences, results of such studies can be regarded as a first step towards identifying respective endosymbiotic species in insects. In the second step of our study, we analysed sequences of specific gene regions for a more detailed phylogeny of selected endosymbiont genera. As a result we identified the presence of *Rickettsia* and “*Candidatus* Nardonella*”* endosymbionts in *Otiorhynchus* spp.. This knowledge is an important step in exploring bacteria-insect associations for potential use in insect pest control.

## Background

It is estimated that more than 65% of insects are associated with symbiotic bacteria, among them *Wolbachia* spp. being the most common genus [1,2]. The range of the symbiotic relationships between insect hosts and bacteria varies from being mutualistic and commensal to a pathogenic one [3-5]. Accordingly, intracellular symbionts in insects are usually referred to as primary or secondary endosymbionts (P- and S-symbionts, respectively), with P-symbionts being obligate for the insect e.g. due to providing nutrients, while S-symbionts have a beneficial but not essential role for host insect survival (for reviews see [3] and [6]). In many insects, endosymbionts are located in specialized organs (referred to as bacteriomes or mycetomes) and their inheritance usually follows a strict vertical transmission from mother to offspring.

Understanding relationships between insect hosts and their endosymbiotic bacteria is not only relevant from an evolutionary point of view, but can also aid in the identification of new targets for insect pest control [7] as well as for biotechnology and biomedicine [3]. Yet, since many of the relevant microorganisms cannot be cultured, their identification and functional characterization was so far difficult or not possible at all. Lately, the accessibility of novel genomic techniques, in particular next generation sequencing (NGS) technologies represent new, cost-efficient and fast strategies to depict microbial diversity without the need for culturing the respective organisms [8]. With these techniques thousands of sequence reads can be analysed in parallel allowing an extensive assessment of bacterial diversity within insects. As a target for bacterial NGS projects, ribosomal DNA genes (rDNA) like the *16S* rDNA, also used for the taxonomic classification of bacterial species [9], have frequently been applied for analysing the bacterial microbial community in metagenomic studies of soil [10,11], mines [12], the deep sea [13] or oral human microflora [14].

In this study, we used high-throughput tag-encoded FLX amplicon pyrosequencing [15] to characterise bacterial communities associated with four different weevil species of the genus *Otiorhynchus* Germar (Coleoptera: Curculionidae). Members of this genus are polyphagous and are regarded as pests of a variety of ornamental and nursery plants worldwide. Their soilborne larvae feed on the host plants’ roots which may be lethal in particular for younger plants or recently transplanted cuttings. Further, feeding damage of adults on the plants foliage may reduce the market value of ornamentals. For these reasons weevils are often controlled by intensive insecticide applications [16]. Moreover, *Otiorhynchus* spp. can serve as a model genus for understanding the evolution of asexual reproduction, since it includes species both reproducing mostly parthenogenetically (like *O. sulcatus* and *O. rugosostriatus*) as well as sexually (like *O. salicicola* and *O. armadillo*) [17,18].

Here, by applying 454 sequencing technology, we show that weevils of the genus *Otiorhynchus* are associated with several endosymbiotic bacteria. This study is the first to report *Rickettsia* and “*Candidatus* Nardonella” endosymbionts - the ancestral endosymbiont of weevils - in *Otiorhynchus* spp.. Identifying endosymbionts in the genus *Otiorhynchus* can expand to our understanding of the evolution of both endosymbiont-host insect interactions as well as of different reproductive strategies of insects and may provide a future basis for novel pest management approaches.

## Results and discussion

### 454 pyrosequencing and identification of endosymbionts in *Otiorhynchus* spp

A total of ~48,000 PCR amplicons were sequenced via GS FLX titanium 454 sequencing, of which ~27,000 reads were assembled after having passed the additional quality controls. These sequences were summarized into 49 consensus sequences (Table 1), representing the total retrieved endosymbiotic bacterial diversity in the four different *Otiorhynchus* species. Sequence abundances of the respective OTUs were different in each weevil species analysed. We expect these differences in sequence abundance within the *16S* rDNA amplicons to reflect the respective bacterial abundances in the sample.

**Table 1:** Endosymbiotic bacterial diversity and abundance in the four analysed *Otiorhynchus* species.

Bacteria from weevil species	GenBank accession No.	Number of reads	% of total reads	Closest phylogenetic match and 16S rDNA accession number	Class
*O. salicicola *(in total 6073 reads)	JN563736	5516	90.83	AB478978, endosymbiont of *Pedicinus obtusus* and AJ245596 endosymbiont of *Camponotus balzanii* (referred to as “*Candidatus* Blochmanni” endosymbionts throughout the text)	γ-Proteobacteria
	JN563737	121	1.99	DQ417336, *Schlegelella aquatica*	β-Proteobacteria
	JN563738	96	1.58	FJ268988, uncultured *Acinetobacter*	γ-Proteobacteria
	JN563739	69	1.14	CU927677, uncultured bacterium	-
	JN563740	48	0.79	FJ534956, uncultured bacterium	-
	JN563741	44	0.72	EF210100, *Enterobacter hormaechei*	γ-Proteobacteria
	JN563742	34	0.56	AY923125, *Streptococcus* sp.	Bacilli
	JN563743	26	0.43	EU464962, uncultured bacterium	-
	JN563744	25	0.41	EU766013, uncultured bacterium	-
	JN563745	23	0.38	FJ393126, uncultured *Bacteroides* sp.	Bacteroidetes
	JN563746	18	0.30	EU721814, uncultured epsilon proteobacterium	ε-Proteobacteria
	JN563747	17	0.28	AY953252, *Prevotella* sp.	Bacteroidetes
	JN563748	15	0.25	FJ799146, bacterium enrichment culture clone LA29	-
	JN563749	11	0.18	EU802152, uncultured bacterium	-
	JN563750	10	0.16	AY568512, *Burkholderia fungorum*	β-Proteobacteria

*O. rugosostriatus *(in total 8584 reads)	JN563751	7800	90.87	AB021128, *Rickettsia* sp.	α-Proteobacteria
	JN563752	396	4.61	EF633744, *Candidatus* Neoehrlichia lotoris	α-Proteobacteria
	JN563753	338	3.94	AB478978, endosymbiont of *Pedicinus obtusus* and AJ245596 endosymbiont of *Camponotus balzanii* (referred to as “*Candidatus* Blochmanni” endosymbionts throughout the text)	γ-Proteobacteria
	JN563754	17	0.20	AB021128, *Rickettsia* sp.	α-Proteobacteria
	JN563755	11	0.13	EF633744, *Candidatus* Neoehrlichia lotoris	α-Proteobacteria
	JN563756	7	0.08	AB021128, *Rickettsia* sp.	α-Proteobacteria
	JN563757	6	0.07	AB021128, *Rickettsia* sp.	α-Proteobacteria
	JN563758	5	0.06	FJ868862, uncultured bacterium	-
	JN563759	4	0.05	GQ845011, *Nevskia* sp.	γ-Proteobacteria

*O. sulcatus* (in total 6412 reads)	JN563760	6358	99.16	AB021128, *Rickettsia* sp.	α-Proteobacteria
	JN563761	35	0.55	EF633744, *Candidatus* Neoehrlichia lotoris	α-Proteobacteria
	JN563762	19	0.30	EF633744, *Candidatus* Neoehrlichia lotoris	α-Proteobacteria

*O. armadillo* (in total 6311 reads)	JN563763	5900	93.49	AB478978, endosymbiont of *Pedicinus obtusus* and AJ245596 endosymbiont of *Camponotus balzanii* (referred to as “*Candidatus* Blochmanni” endosymbionts throughout the text)	γ-Proteobacteria
	JN563764	60	0.95	FJ823944, uncultured *Comamonas* sp.	β-Proteobacteria
	JN563765	54	0.86	FJ868862, uncultured bacterium	-
	JN563766	43	0.68	FJ823944, uncultured *Comamonas* sp.	β-Proteobacteria
	JN563767	35	0.55	FJ544375, *Comamonas aquatica*	β-Proteobacteria
	JN563768	31	0.49	EU560802, uncultured bacterium	-
	JN563769	23	0.36	DQ407746, primary endosymbiont of *Liposcelis decolor*	-
	JN563770	21	0.33	DQ469223, uncultured bacterium	-
	JN563771	21	0.33	GQ845011, *Nevskia* sp.	γ-Proteobacteria
	JN563772	20	0.32	DQ860049, uncultured bacterium	-
	JN563773	11	0.17	AF006670, *Shewanella putrefaciens*	γ-Proteobacteria
	JN563774	11	0.17	X82133, *Shewanella putrefaciens*	γ-Proteobacteria
	JN563775	11	0.17	EU801479, uncultured bacterium	-
	JN563776	10	0.16	EF019306, uncultured proteobacterium	-
	JN563777	9	0.14	AY953252, *Prevotella* sp.	Bacteroidetes
	JN563778	8	0.13	EU464962, uncultured bacterium	-
	JN563779	8	0.13	EU536078, uncultured bacterium	-
	JN563780	8	0.13	GQ068015, uncultured bacterium	-
	JN563781	8	0.13	L16490, *Porphyromonas asaccharolytica*	Bacteroidetes
	JN563782	8	0.13	AY351787, uncultured marine bacterium	-
	JN563783	6	0.10	EF648074, uncultured *Azoarcus* sp.,	β-Proteobacteria
	JN563784	5	0.08	EF648074, uncultured *Azoarcus* sp.,	β-Proteobacteria

Only the closest relatives and their *16S* rDNA accession numbers (see additional file 1: *16S* rDNA gene-based phylogeny of endosymbionts in four different *Otiorhynchus* spp. larvae) are mentioned.

In addition to the most abundant reads, which belonged either to the genus *Rickettsia* or were similar to “*Candidatus* Blochmannia” bacteria and endosymbionts of the lice *Pedicinus obtusus* and *P. badii*, numerous reads with low sequence frequency were detected (Table 1). Indeed, we can not fully exclude the possibility that these sequences of putative rare endosymbionts are rather artefacts e.g. due to PCR contaminations.

### Phylogenetic analysis of *Otiorhynchus* spp. endosymbionts

**Figure 1:**
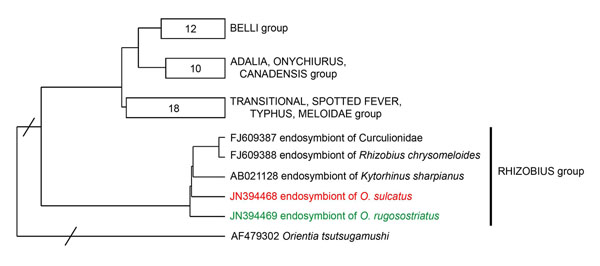
**Neighbour joining tree of *Rickettsia* endosymbionts using sequences of *16S* rDNA.** Sequences obtained in the present study are coloured and phylogenetic groups were constructed according to Weinert et al [22]. The amount of sequences included in the groups are indicated by numbers. Branch lengths were reduced in two positions (marked with diagonal slashes).

**Figure 2:**
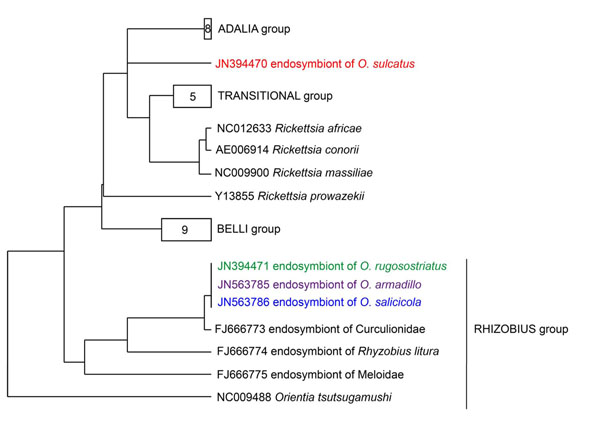
**Neighbour joining tree of *Rickettsia* endosymbionts using sequences of *coxA* gene.** Sequences obtained in the present study are coloured. Sequences were combined in groups according to Weinert et al [22]. The amount of sequences included in the groups are indicated by numbers.

**Figure 3:**
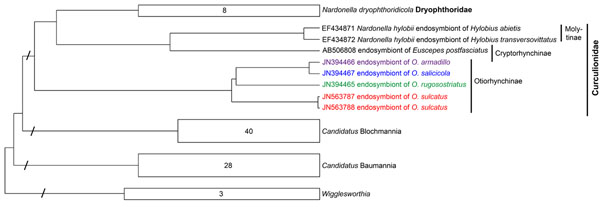
**Neighbour joining tree of *“Candidatus* Nardonella” endosymbionts using sequences of *16S* rDNA.** Sequences obtained in the present study are coloured. Branch lengths were reduced in four positions (marked with diagonal slashes). The amount of sequences included in the groups are indicated by numbers.

Phylogenetic analysis of 454 sequence data was performed to establish the relationship of the partial *16S* rDNA sequences to each other and to related sequences gained from public databases. Among all studied weevil species, *O. sulcatus* showed the lowest bacterial endosymbiotic diversity (Table 1). The vast majority of sequences in *O. sulcatus* (~99% of the total reads) and *O. rugosostriatus* (~91% of the total reads) belonged to the genus *Rickettsia* (see additional file 1: *16S* rDNA gene-based phylogeny of endosymbionts in four different *Otiorhynchus* spp. larvae, Table 1). Bacteria similar to the endosymbionts of the lice *Pedicinus obtusus* and *P. badii *[19,20] and the genus “*Candidatus* Blochmannia” were dominant in *O. salicicola* (~91% of the total reads) and *O. armadillo* (~93% of the total reads) (see additional file 1: *16S* rDNA gene-based phylogeny of endosymbionts in four different *Otiorhynchus* spp. larvae, Table 1). These bacteria were also found in a less dominant manner in *O. rugosostriatus* (~4% of the total reads). To determine the phylogenetic position of *Rickettsia* and putative “*Candidatus* Blochmannia” like endosymbionts detected via 454 pyrosequencing in a more precise way, genus specific primers [21,22] were used to amplify a ~750 bp fragment of the *Rickettsia* and “*Candidatus* Blochmannia” specific *16S* rDNA and a ~800 bp fragment of the *Rickettsia* cytochome C subunit I (*coxA*) gene, respectively. Phylogenetic analysis of these sequences placed the *Otiorhynchus* spp. specific *Rickettsia* into a new clade within the genus *Rickettsia* (Figure 1 and 2). Sequences gained by using “*Candidatus* Blochmannia” specific primers were grouped within the clade of “*Candidatus* Nardonella*”* bacteria, which are closely related to “*Candidatus* Blochmannia” endosymbionts (Figure 3). Accordingly, the additional analysis of these endosymbionts using gene specific primers revealed for the first time the presence of *Rickettsia* and “*Candidatus* Nardonella” bacteria within the genus *Otiorhynchus* spp..

### Phylogenetic analysis and putative biological function of *Rickettsia* endosymbionts

In the parthenogenetically reproducing species *O. sulcatus* and *O. rugosostriatus*, *Rickettsia* endosymbionts were the most dominant group found via 454 pyrosequencing. By using *Rickettsia* specific primers for the *16S* rDNA and the *coxA* gene these results were strengthened, however, a fragment of the *Rickettsia* specific *coxA* gene was also amplified in *O. armadillo* and *O. salicicola*, respectively, while 454 pyrosequencing previously indicated that these two species were missing *Rickettsia* endosymbionts (Table 1). Yet, at the same time it was not possible to amplify the *Rickettsia* specific *16S* rDNA fragment in the same two species. We thus suppose that the *coxA* gene sequence is rather conserved among bacteria and may not be adequate for precise species determination. Supplementary sequence analysis of a range of additional bacterial genes may resolve this issue.

Phylogenetic analysis of the *Rickettsia* endosymbiontic *16S* rDNA and *coxA* gene fragments amplified from *Otiorhynchus* spp. revealed the relatedness to the rhizobius and/or adalia *Rickettsia* group as defined by Weinert et al [22]. These subgroups contain *Rickettsia* bacteria identified in various beetles, including members of the Curculionidae [22]. *Rickettsia* endosymbionts act as male-killing agents in leaf mining beetles and ladybirds [23,24] and play an essential role in the early development of the oocyte and egg production in parthenogenetic book lice [25,26]. Thus it could be speculated that *Rickettsia* endosymbionts may also manipulate host reproduction in *Otiorhynchus* species.

### Phylogenetic analysis and putative biological function of “*Candidatus* Nardonella” endosymbionts

454 pyrosequencing detected endosymbionts similar to “*Candidatus* Blochmannia” and bacterial endosymbionts of the lice *Pedicinus obtusus* and *P. badii* in *O. armadillo*, *O. salicicola* and to a lesser extent in *O. rugosostriatus*. The presence of these putative “*Candidatus* Blochmannia” like bacteria was verified in these species by using primers specific for the “*Candidatus* Blochmannia” *16S* rDNA [21], which indicated that the obtained sequences are similar to “*Candidatus* Nardonella”. In addition, a fragment of the same size and sequence was also amplified in *O. sulcatus*, even though 454 pyrosequencing did not reveal the presence of these bacteria in this weevil species (Table 1). “*Candidatus* Nardonella” bacteria are often localized in the bacteriome whereas *Rickettsia* endosymbionts may infect as well different tissues. As we used whole larvae for DNA extraction, the amount of overall isolated DNA might have been lower for “*Candidatus* Nardonella” than for *Rickettsia*. Therefore we assume that respective bacterial DNA might have not been amplified in *O. sulcatus* with the universal primers used for 454 pyrosequencing due to competition for PCR reagents with taxa such as *Rickettsia*, having a higher template abundance [27]. However, these results also demonstrate that studies using 454 pyrosequencing can be regarded as a first step towards identifying respective endosymbiotic species in insects, but that for a detailed phylogeny and a more comprehensive insight into endosymbiont-insect-associations, the amplification of specific gene regions is still indispensable.

Phylogenetic analysis of the putative “*Candidatus* Blochmannia” specific *16S* rDNA sequence amplified from the four studied *Otiorhynchus* weevils showed a close relatedness of these bacteria to the genus “*Candidatus* Nardonella”. Sequences generated in the present study build a separate branch next to endosymbionts from molytine, cryptorhynchine and dryophthorid weevils [28-30] (Figure 3). The biological function of “*Candidatus* Nardonella” endosymbionts in their host weevils is unknown so far, except for the cryptorhynchine West Indian sweet potato weevil, *Euscepes postfasciatus.* Within this species “*Candidatus* Nardonella*”* endosymbionts are involved in growth and development of the host weevil [31].

### Implications and future directions of endosymbiosis in *Otiorhynchus* spp

**Figure 4:**
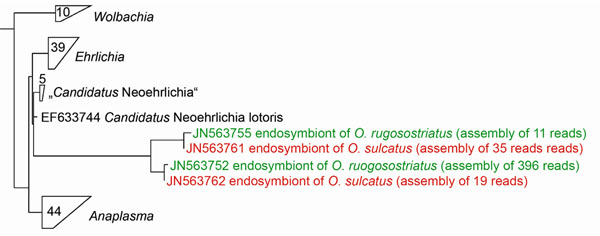
**Phylogenetic analysis of endosymbionts under “*Candidatus* Neoehrlichia” subregion in *Otiorhynchus* spp.** The tree represents the “*Candidatus* Neoehrlichia” subregion of the complete tree (see additional file 1: *16S* rDNA gene-based phylogeny of endosymbionts in four different *Otiorhynchus* spp. larvae) and was constructed by using parsimony algorithm. Sequences obtained in the present study are coloured. The amount of sequences included in the groups of *Wolbachia*, *Ehrlichia*, „*Candidatus* Neoehrlichia” and *Anaplasma* are indicated by numbers.

For several *Otiorhynchus* species, an association with bacteria of the genus *Wolbachia* has been proven in previous studies [32-34]. *Wolbachia* cause several reproductive alterations in insects, including cytoplasmic incompatibility, feminization of genetic males or parthenogenesis [35]. In *Otiorhynchus* species *Wolbachia* are assumed to rather play a role in normal development of e.g. *O. sulcatus* eggs [34] rather than in the evolution of parthenogenesis or polyploidy [32,33,36]. Unexpectedly, in the present 454 pyrosequencing approach, none of the bacterial sequence reads obtained from four different *Otiorhynchus* spp. weevil larvae corresponded to *Wolbachia.* Instead, bacterial sequences similar to “*Candidatus* Neoehrlichia”, a close relative to *Wolbachia*, were found in however low frequencies in *O. sulcatus* (~1% of the total reads) and *O. rugosostriatus* (~5% of the total reads) (Table 1, Figure 4). Species of that genus are known as tick-borne bacterial pathogens [37] and have been isolated from raccoons and rats [38,39] but their biological function in insects is unclear so far. As the presence of different *Wolbachia* strains may differ within a given species between geographical regions [40] further studies are required using *Wolbachia* specific PCR primers to shed light on the prevalence and distribution of *Wolbachia* within *Otiorhynchus* species and between populations, respectively.

Recent microbiological characterization of bacterial endosymbionts in the Curculionoidea of the family Molytinae and Dryophthoridae has demonstrated that endosymbiosis with “*Candidatus* Nardonella” bacteria is ~125 Myr old in curculionids and is most of the times evolutionary stable, except for a few clades where respective endosymbionts have been lost and were replaced by different microbes during evolution (endosymbiont replacement; [29]). Our study broadens the range of weevils associated with “*Candidatus* Nardonella” endosymbionts and indicates a benefit for *Otiorhynchus* weevils due to the long-lasting bacterial inheritance.

In a number of weevil species it has been shown that endosymbionts are frequently found within specialized host cells (so-called bacteriocytes) sometimes forming a distinctive organ, the bacteriome, which is often associated with the larval midgut [29,30,41-43]. As Buchner [44] has described a bacteriome in *Otiorhynchus* spp., we assume that the four *Otiorhynchus* species analysed in the present study also harbour their endosymbiotic bacteria intracellularly in a bacteriome. However, this assumption has to be confirmed via microscopic examinations of the respective organs.

For a couple of insects and their associated microorganisms it has been shown, that endosymbiotic bacteria are known to be involved in protecting their host insect against natural antagonists such as predators and pathogens or are even implicated in insecticide resistance mechanisms (for a review see Zindel et al [45]). Moreover, particularly obligatory endosymbionts are essential for central functions of their host insect [3]. Accordingly, endosymbiotic bacteria are an interesting target for direct or indirect manipulation, thus offering new possibilities for designing insect control strategies [45-47]. Identification of respective endosymbiotic organisms of the target insect is an important step in exploring these associations for potential use in insect pest control. Thanks to the agar-based artificial diet for rearing of *O. sulcatus *[48], physiological, nutritional and reproductive studies will be carried out to analyse the respective effects of symbionts on the host development and reproduction.

## Conclusions

In this study, endosymbiotic bacterial diversity in weevil larvae was assessed via multitag 454 pyrosequencing of a bacterial *16S* rRNA fragment. Pyrosequencing is therefore a promising, fast and economic alternative to other culture-independent methods in metagenomics like DGGE (Denaturing Gradient Gel Electrophoresis) or SSCP (Single Strand Conformation Polymorphism), which have been used in bacterial community studies of the red turpentine beetle [49] or for diversity assessment of gut microbiota in bees [50], respectively. However, as 454 pyrosequencing generates only quite short sequences, results of such studies can just be regarded as a first step towards identifying respective endosymbiotic species in insects. Accordingly, a subsequent analysis of sequences of specific gene regions of selected endosymbiont genera detected via 454 pyrosequencing revealed the presence of endosymbionts of the genera *Rickettsia* and “*Candidatus* Nardonella” in *Otiorhynchus* spp.. Further studies are now required to clarify the biological function of these endosymbiotic bacteria in *Otiorhynchus* spp. and their potential as novel targets for weevil pest control.

## Materials and methods

### Insect rearing, bacterial DNA extraction and species determination of larvae

All experiments were performed with four different *Otiorhynchus* species (*O. sulcatus*, *O. rugosostriatus*, *O. salicicola* and *O. armadillo*) collected in the field and kept in the laboratory until egg deposition. During that period of time weevils were fed with leaves of *Prunus* sp., *Potentilla* sp. or *Fragaria* sp.. Freshly laid weevil eggs (at most 10 days old) were collected and surface sterilized according to the method developed by Hosokawa et al [51]. The eggs were air dried under the clean bench and transferred individually with sterile featherweight forceps in Petri dishes filled with sterile TSA (40,0 g/l Difco^TM ^Tryptic Soy Agar, pH 7.3 ± 0.2; Voigt Global Distribution Inc, Lawrence, Kansas). In order to enlarge the contact of egg and TSA agar and to check the success of surface sterilisation, eggs were rolled several times over the agar plate. For further analysis only eggs with no bacterial growth on TSA were included. Eggs were kept usually at 21-24°C until eclosion. Freshly emerged larvae (approximately 24-72 hours old) without egg material were individually collected from the TSA agar plates, and were stored frozen at -80°C until further processing. Total metagenomic DNA (~20-40 ng/µl DNA per larva) was extracted from the complete larvae using the MasterPure^TM^ DNA Purification Kit (Epicentre^®^ Biotechnologies, Madison, Wisconsin). Taxonomic identity of each larva was confirmed according to a diagnostic PCR-RFLP pattern of the COII region [52]. For metagenomic analysis seven individuals of each *Otiorhynchus* species were included.

### Bacterial 16S rDNA PCR amplification and 454 pyrosequencing

Universal bacteria primers (fwd: 5’-MGAGTTTGATCCTGGCTCAG-3’ and rev: 5’-GCTGCCTCCCGTAGGAGT-3’; [53]), amplifying an approximately 450 bp fragment of the *16S* rDNA, were used in the present study. These primers are covering the V1-V2 regions of the *16S* rDNA gene and showed good phylogenetic resolution from phylum to family level in a recent study by Hamp et al [53]. Primers were modified by the addition of a GS FLX Titanium Key-Primer A and B (A: CGTATCGCCTCCCTCGCGCCA and B: CTATGCGCCTTGCCAGCCCGC), a four-base library “key” sequence (TCAG) and a multiplex identifier (MID) sequence specific to each *Otiorhynchus* species. The MID sequences (forward/reverse) were as follows for the respective weevil species: *O. salicicola* (ATCGCG / CGCGAT), *O. rugosostriatus* (ATAGCC / GGCTAT), *O. sulcatus* (CCATAG / CTATGG) and *O. armadillo* (CTTGAG / CTCAAG). PCR reaction mixture consisted of 0.1 µl of Phire^®^ Hot Start II DNA Polymerase (Finnzymes Oy, Espoo, Finland), 0,2 mM dNTPs (Metabion, Martinsried, Germany), 10 pmol primers and 40-80 ng of DNA template in a final volume of 20 µl. The PCR parameters (C1000^TM^ Thermal Cycler, Bio-Rad Laboratories GmbH, München, Germany) were 95°C for 3 min followed by 35 cycles of 93°C for 60 s, 50°C for 60 s and 72°C for 70 s. A final extension step at 72°C for 5 min was added. An aliquot of 4 µl of each PCR product was checked for correct size (~450 bp) on a 1% agarose gel and was afterwards purified with HiYield PCR Clean-up/Gel Extraction Kit (Süd-Laborbedarf GmbH, Gauting, Germany). Bacterial *16S* rDNA PCR products generated from all 28 *Otiorhynchus* individuals were mixed at equal molar concentrations according to species, and next generation 454 pyrosequencing was performed commercially (LGC Genomics GmbH, Berlin, Germany). The GenBank accession numbers for sequences obtained via 454 pyrosequencing are listed in Table 1.

### Sample assignment and analysis of 454 sequencing data

Sequence reads were assembled independently by Geneious Pro Version 5.0 [54] and WiMSeEx (Window Match Seed Extension)-Algorithm (unpublished). Results of both procedures for diversity and sequence identity were compared. Only high quality reads that did accurately match the four-base library “key” sequence (TCAG) and the multiplex identifier (MID) sequence were used for Geneious Pro assembly. Geneious Pro assembly was performed with medium sensitivity, a maximum of 120 contigs and default settings. Consensus sequences were extracted manually from all contigs. WiMSeEx assembly was performed for each tag with all raw data reads and the following parameters: minimum seed size: 200 bp, window size: 60 bp. The four-base identifier and 20 bp of the primer were chosen for seed detection. Each assembly run was stopped by reaching 500 kb sequence data. Resulting sequences of both procedures were then aligned independently using MAFFT version 5 [55] and consensus sequences were extracted manually from clustered sequences and redundant sequence data were removed. Afterwards the sequence identifier and the primer sequence were eliminated from each consensus sequence. All consensus sequences extracted from Geneious Pro contigs were found in the WiMSeEx consensus sequences assembly data and vice versa.

### Amplification of selected genes of most dominant endosymbionts

For accurate phylogenetic analysis of the most dominant endosymbionts in *Otiorhynchus* spp., specific *16S* rDNA and cytochrome C oxidase subunit I (*coxA*) primers for the genus *Rickettsia *[22] as well as *16S* rDNA primers for “*Candidatus* Blochmannia*”* bacteria [21] were used for amplification of the respective sequences from 2-4 *Otiorhynchus* individuals per species. PCR reactions were set up in a final volume of 20 µl consisting of 0.1 µl of Phire^®^ Hot Start II DNA Polymerase (Finnzymes Oy, Espoo, Finland), 0.25 mM dNTPs (Fermentas GmbH, St. Leon-Rot, Germany), 10 pmol primers and 40-80 ng of DNA template. The PCR parameters (C1000^TM^ Thermal Cycler, Bio-Rad Laboratories GmbH, München, Germany) were 95°C for 2 min followed by 40 cycles of 95°C for 30 s, 55°C for 30 s and 72°C for 1 min. A final extension step at 72°C for 10 min was added. An aliquot of 4 µl of each PCR product was checked for correct size on a 1% agarose gel and was afterwards purified with HiYield PCR Clean-up/Gel Extraction Kit (Süd-Laborbedarf GmbH, Gauting, Germany). Direct sequencing of the resulting PCR product was performed commercially (LGC Genomics GmbH, Berlin, Germany). As we did not detect any bacterial sequence variation within one weevil species (except for *O. sulcatus* and the *16S* rDNA amplified with “*Candidatus* Blochmannia” specific primers), only one sequence per *Otiorhynchus* species and gene region was submitted to GenBank (accession numbers JN394465-JN394471, JN563785-JN563788).

### Phylogenetic analysis

Consensus sequences gained from 454 pyrosequencing were included into an alignment of more than 260,000 (SSURef_102_SILVA_NR_99_18_02_10_opt.ARF) bacterial *16S* rDNA sequences [56] and best positions in the resulting phylogenetic tree were found including all nucleotides (positions) from the 454 assemblies using the Parsimony algorithm of the ARB 5.1 software package [57]. The here presented trees are subregions of the complete tree (see additional file 1: *16S* rDNA gene-based phylogeny of endosymbionts in four different *Otiorhynchus* spp. larvae) including the sequences assembled from the 454 sequencing approach reported in this paper and the most similar sequences available from public databases. More distantly related or unrelated sequences were included in the calculation but are not shown.

Additional *16S* rDNA sequences amplified with specific primers for “*Candidatus* Blochmannia” and *Rickettsia* endosymbionts were included in the above mentioned alignment and a Neighbour joining analysis was inferred using the Neighbour joining algorithm included in the software package ARB 5.1 like described above. In addition, sequences of part of the *coxA* gene amplified in *Otiorhynchus* spp. were included in an alignment of sequences used by Weinert et al [22] and a Neighbour joining tree was calculated accordingly.

## Authors’ contributions

JH and AR conceived the study design; JH performed sample collection and template preparation for pyrosequencing analysis; JH, SS, and MP performed phylogenetic analysis, and all authors contributed to the writing of the manuscript.

## Competing interests

The authors declare that they have no competing interests.

## Supplementary Material

Additional file 1 ***16S* rDNA gene-based phylogeny of endosymbionts in four different *Otiorhynchus* spp. larvae.** Sequences obtained in the present study are coloured and accession numbers of *16S* rDNA sequences are shown for related bacterial species. More distantly related or unrelated sequences are not shown. Sequences from this work were added using the parsimony algorithm. This tree results from a phylogenetic calculation including more than 26,0000 bacterial *16S* rDNA sequences. Only the nearest relatives are shown in this tree. Click here for file
